# TFAP2B overexpression contributes to tumor growth and progression of thyroid cancer through the COX-2 signaling pathway

**DOI:** 10.1038/s41419-019-1600-7

**Published:** 2019-05-21

**Authors:** Xiaoyan Fu, Huayong Zhang, Zhipeng Chen, Zhongyuan Yang, Dingbo Shi, Tianrun Liu, Weichao Chen, Fan Yao, Xuan Su, Wuguo Deng, Miao Chen, Ankui Yang

**Affiliations:** 1 Sun Yat-sen University Cancer Center; State Key Laboratory of Oncology in South China; Collaborative Innovation Center of Cancer Medicine, Guangzhou, Guangdong China; 2 Department of Thyroid and Breast Surgery, the Fifth Affiliated Hospital of Sun Yat-Sen University, Zhuhai, China; 3 Department of Otorhinolaryngology Head and Neck Surgery, The Sixth Affiliated Hospital of Sun Yat-sen University, Guangzhou, Guangdong China

**Keywords:** Head and neck cancer, Head and neck cancer

## Abstract

Thyroid cancer is commonly seen in the clinic with a rapidly increasing incidence globally. COX-2 overexpression correlates with the pathologic type of thyroid carcinoma, and it has been suggested that COX-2 overexpression is associated with a poor prognosis. However, little is known about its upstream regulatory mechanism. Bioinformatics suggested that transcription factor AP-2 beta (TFAP2B) might specifically bind to the COX-2 promoter, which was confirmed by biotin-labeled COX-2 promoter pulldown and luciferase reporter assays. We performed western blot and immunohistochemical staining to detect the expression of TFAP2B/COX-2 in thyroid cancer tissues (T) and the matched adjacent noncarcinoma tissues (ANT), and investigated the relationship between TFAP2B/COX-2 expression and clinical pathological factors in thyroid cancer patients. Afterward, MTS, colony formation, cell-apoptosis assay, transwell-invasion and scratch assays were performed to examine the proliferation, apoptosis, invasion, and migration of thyroid cancer cells with TFAP2B knocked down or overexpressed. The mouse xenograft experiment was performed to study in vivo the proliferation of thyroid cancer cells with TFAP2B knocked down or overexpressed. We found that TFAP2B bound to the promoter of COX-2 to activate its expression. Western blot and immunohistochemistry showed that TFAP2B/COX-2 was highly expressed in thyroid cancer, and high TFAP2B and COX-2 expression was associated with aggressive clinicopathological features in thyroid cancer. TFAP2B mediated thyroid cancer cell proliferation, apoptosis, invasion, and migration via the COX-2 signaling pathway in vitro and in vivo. TFAP2B bound to the promoter of COX-2 to activate its expression, indicating that TFAP2B is a critical regulatory molecule in the COX-2 signaling pathway that promoted tumor progression in thyroid cancer.

## Introduction

As an endocrine malignancy, thyroid cancer is commonly seen in the clinical, and has rapidly increased in global incidence in recent decades^1^. The average annual increase in thyroid cancer incidence (6.6%) between 2000 and 2009 is the highest among all cancers in the United States^2^. Although thyroid cancers such as papillary thyroid cancer (PTC) have favorable prognosis and a low death rate, a certain number of patients develop a more aggressive forms that is unresponsive to radioactive iodine and chemotherapy^3^, resulting in increased incurability and patient morbidity and mortality, which are associated with large societal healthcare burdens. Therefore, gaining insight into the mechanism of thyroid cancer development and identifying novel tumor molecular markers will be helpful for the effective identification of thyroid cancer patients with a high risk of recurrence and metastasis.

Cyclo-oxygenase-2 (COX-2) is a key enzyme in eicosanoid biosynthesis. In previous studies, upregulated COX-2 expression has been observed in a number of malignant tumors, such as breast cancer and colon cancers^4,5^. The overexpression of COX-2 is related to prostaglandin-stimulating angiogenesis and proliferation, which can promote cell invasion and the development of metastases^6^. In summary, by participating in promoting cell proliferation, inhibiting cell apoptosis, and enhancing angiogenesis, COX-2 can promote carcinogenesis and cancer progression^7–10^. It has been shown that COX-2 can be modulated by cleavage- and polyadenylation-specific factor 4 (CPSF4) as a transcriptional regulator in lung cancer^11^. Previous studies have confirmed that COX-2 overexpression correlates with the pathologic type of thyroid carcinoma and have suggested that COX-2 overexpression is associated with a poor prognosis^12,13^. However, we do not know much about its upstream regulatory mechanism.

Bioinformatics suggest that TFAP2B may specifically bind to the COX-2 promoter, modulating the tumorigenesis and development of cancer. To confirm this prediction in thyroid cancer, we investigated the association between thyroid tumorigenesis and TFAP2B/COX-2 expression and determined whether TFAP2B modulated COX-2 to promote thyroid cancer progression.

TFAP2 is a family of transcription factors that consists of the following members: TFAP2A, TFAP2B, TFAP2C, TFAP2D, and TFAP2E^14^. TFAP2 plays an important role in regulating various aspects of tissue development during embryogenesis^15^. In mice, TFAP2B deletion has been demonstrated to cause congenital polycystic kidney disease, and several point mutations in the TFAP2B coding region are associated with Char syndrome, a human autosomal dominant disorder^16–18^. In a mouse experimental model, the loss of TFAP2 transcription leads to cell proliferation and induces premature differentiation or apoptosis during tissue development. During embryogenesis, the TFAP2 family plays important roles in controlling the balance between proliferation and differentiation, and the TFAP2 family has been implicated in cell growth, differentiation, apoptosis, and especially carcinogenesis in recent years^19^. Previous studies showed that TFAP2B was demonstrated to be highly expressed in human lung adenocarcinoma and it was positively correlated with the poor prognoses of lung adenocarcinomas (*P* < 0.001), knockdown of TFAP2B inhibited cell growth and induced apoptosis in lung adenocarcinoma cells in vitro and in a lung cancer subcutaneous xenograft model by simultaneously regulating multiple signaling pathways, such as the ERK/p38, VEGF/PEDF, and caspase-dependent pathways^20^. TFAP2A, TFAP2B, and TFAP2C are expressed in breast tissue and are thought to coordinate human epidermal growth factor receptor 2 (HER2) and estrogen receptor (ER)^19^. Because the oncogene TFAP2B has been studied for several years, it has been clarified that TFAP2B expression can be associated with cancer prognosis^21,22^. For example, gene-expression profiling has implicated TFAP2B in breast cancer (BC), and knockdown of TFAP2B diminished proliferation of lobular BC cell lines in vitro, indicating that TFAP2B controls tumor cell proliferation in slow-growing BC subtype^22^. However, we do not know much about its transcriptional regulatory mechanism in tumorigenesis.

We examined the expression of TFAP2B/COX-2 at the protein level in thyroid cancer cell lines and tumor tissues. We knocked down and overexpressed TFAP2B to evaluate the effect of TFAP2B on thyroid cancer cell growth, metastasis, and invasion, and we further elucidated the underlying molecular mechanisms involved in modulating COX-2. We also analyzed thyroid cancer specimens in a tissue array and evaluated the prognostic predictive value of TFAP2B/COX-2 in thyroid cancer by combining the information with the clinical data. A thyroid cancer xenograft model was used to confirm thyroid tumorigenesis with TFAP2B/COX-2 expression. We clearly substantiated the biological role of TFAP2B/COX-2 in thyroid cancer and suggest that it could serve as a novel therapeutic and diagnostic target for thyroid cancer.

## Results

### Bioinformatics analysis identified TFAP2B as a COX-2 promoter-binding protein

**Fig. 1:**
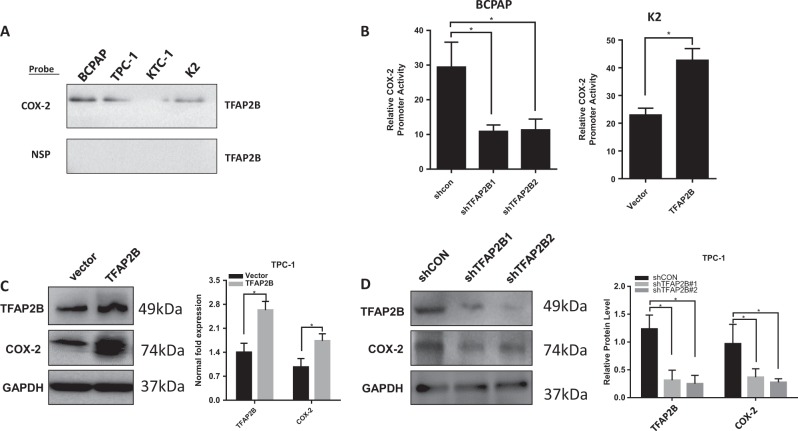
TFAP2B was identified as a COX-2 promoter-binding protein in thyroid cancer cells. **a** Binding of TFAP2B on the 5′-biotin-labeled COX-2 promoter probe or a nonspecific probe (NSP) was detected by western blot using anti-TFAP2B antibody in thyroid cancer cell lines (BCPAP, TPC-1, KTC-1, K2). **b** Relative COX-2 promoter activity in TFAP2B knockdown BCPAP cells and TFAP2B overexpress K2 cells (*n* = 3). **c** COX-2 protein expression was upregulated in TFAP2B overexpressed TPC-1 cells, and relative COX-2 mRNA level was upregulated in TFAP2B overexpressed TPC-1 cells (*n* = 3). **c** COX-2 protein expression was downregulated in TFAP2B knockdown TPC-1 cells, and relative COX-2 mRNA level was downregulated in TFAP2B knockdown TPC-1 cells (*n* = 3)

To clarify the modulated mechanism by which COX-2 acts in thyroid cancer, we further used bioinformatics to determine what can bind to its promoter. By querying the TRANSFAC database, we found that TFAP2B may be a binding protein that combines with COX-2 promoter regions. The binding capacity of TFAP2B to COX-2 was predicted by the JASPRR database, and there are 13 possible binding sites for TFAP2B in the COX-2 promoter region (Table 1). Previously, a 479-bp biotin-labeled double-stranded DNA probe corresponding to the 5′-flanking sequence of the COX-2 gene promoter region was synthesized^23^. To validate the probable prediction of the interaction between TFAP2B and the COX-2 promoter, we pulled down the nuclear proteins bound to the COX-2 promoter in thyroid cancer cells using the 5′-biotin-labeled COX-2 promoter probe or a nonspecific probe (NSP) and streptavidin–agarose beads and used western blotting to detect TFAP2B in the nuclear protein/DNA complex with a TFAP2B-specific antibody. High levels of TFAP2B were bound to the COX-2 promoter probe in thyroid cancer cells (K2, TPC-1, KTC-1, and BCPAP) (Fig. 1a, upper panel). We also detected the other TFAP2 family members (TFAP2A, TFAP2C) using a COX-2 promoter probe in thyroid cancer cell lines as described above, and we did not find any other TFAP2 family members bind to the COX-2 promoter probe. We investigated the effect of TFAP2B on COX-2 promoter activity and gene expression in this study. The luciferase reporter assay showed that COX-2 promoter activity obviously decreased when TFAP2B was knocked down in BCPAP cells compared with the nonspecific shRNA control (shNC) group and increased when TFAP2B overexpressed in K2 cell line (Fig. 1b). Furthermore, overexpression of TFAP2B promoted COX-2 expression at the protein and RNA levels in TPC-1 cells, while knockdown of TFAP2B inhibited COX-2 expression in TPC-1 cells (Fig. 1c, d).

**Table 1:** Bioinformatics analysis identified TFAP2B as a COX-2 promoter-binding protein

13 putative sites were predicted with these settings (80%) in sequence named hg38_refGene_NM_000963							
Model ID	Modle name	Score	Relative score	Start	End	Strand	Predicted site sequence
MA0811.11	TFAP2B	14.597	0.974299637132756	1066	1077	−1	tgccttaaggca
MA0811.11	TFAP2B	14.597	0.974299637132756	1066	1077	1	tgccttaaggca
MA0811.11	TFAP2B	4.050	0.807030550489048	1568	1579	−1	ggtccgggggcg
MA0811.11	TFAP2B	5.440	0.829075115809495	1568	1579	1	cgcccccggacc
MA0811.11	TFAP2B	5.319	0.82715612846865	1576	1587	−1	ggccctgaggtc
MA0811.11	TFAP2B	6.752	0.849882647968075	1576	1587	1	gacctcagggcc
MA0811.11	TFAP2B	6.846	0.85137343152212	1676	1687	−1	tgcccgagcgct
MA0811.11	TFAP2B	6.593	0.847361003445808	1676	1687	1	agcgctcgggca
MA0811.11	TFAP2B	6.483	0.845616469499585	1727	1738	−1	cgccccaggcgc
MA0811.11	TFAP2B	5.948	0.83713169076114	1727	1738	1	gcgcctggggcg
MA0811.11	TFAP2B	7.551	0.862554308177454	1728	1739	−1	ccgccccaggcg
MA0811.11	TFAP2B	5.804	0.83484793723154	1728	1739	1	cgcctggggcgg
MA0811.11	TFAP2B	4.690	0.81718056617616	1861	1872	1	ccccaccgggct

Comment: This type of analysis has a high sensitivity but abysmal selectivity. In other words: while true functional will be detected in most cases, most predictions will correspond to sites bound in vitro but with no function in vivo. A number of additional contraionts of the analysis can improve the prediction; phylogenetic footprinting is the most common. We recommend using the ConSites service, which uses the JASPAR datasets

The review^36^ gives a comprehensive overview of transcription binding site prediction

The binding capacity of TFAP2B to COX-2 was predicted by the JASPRR database, and there are 13 possible binding sites for TFAP2B in the COX-2 promoter region

### TFAP2B/COX-2 is highly expressed in thyroid cancer

**Fig. 2:**
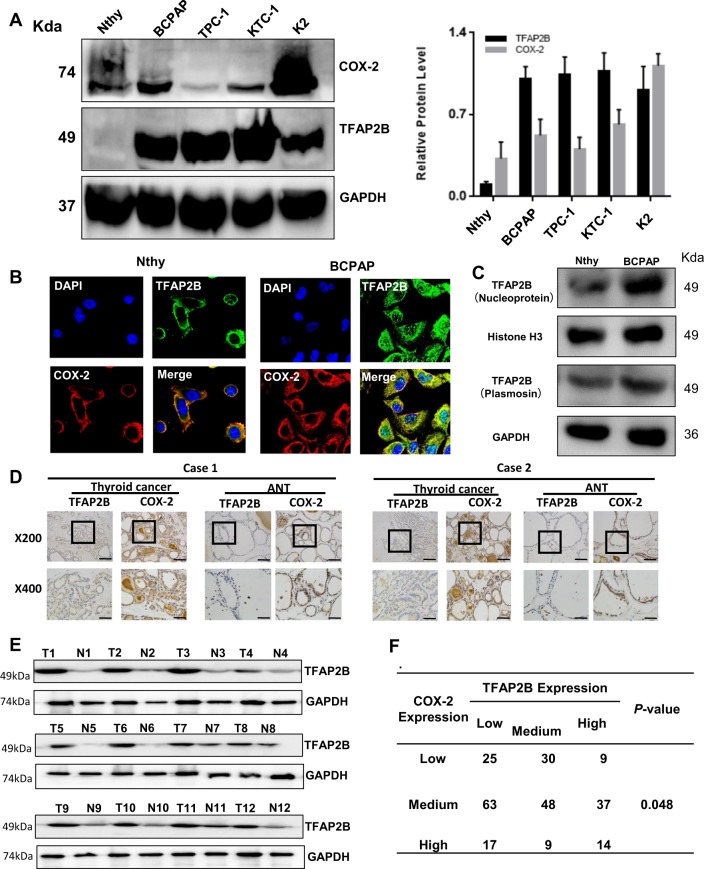
TFAP2B/COX-2 is highly expressed in thyroid cancer. **a** The expression of the total TFAP2B/COX-2 proteins in normal thyroid gland cell (Nthy-ori 3–1) and thyroid cancer cells (BCPAP, TPC-1, KTC-1, and K2) were analyzed by western blot, and the relative protein level in different thyroid cancer cells is shown in **a** (right) (*n* = 3). **b** Protein subcellular localization of TFAP2B/COX-2 was examined by confocal microscopy analysis in BCPAP and Nthy-ori 3–1 cell line. **c** Both nucleic and cytoplasmic expression of TFAP2B can be detected in Nthy-ori-3-1 and BCPAP cell line. **d** The immunohistochemical results demonstrated the representative images of TFAP2B and COX-2 expression in thyroid cancer tissues and adjacent noncarcinoma tissues (ANTs) (x200 upper, x400 down). **e** The western blot results demonstrated TFAP2B and COX-2 expression in thyroid cancer tissues and ANTs. **f** The correlation between the expression of TFAP2B and COX-2 in human thyroid tissues from 252 patients (*P* < 0.05)

Then, we examined the expression of TFAP2B/COX-2 at the protein level in human thyroid normal cell lines (Nthy-ori-3-1), and thyroid cancer cell lines (TPC-1, KTC-1, BCPAP, K2) by western blotting (Fig. 2a), the amount of transcripts of TFAP2B and COX-2 in each cell is shown in Supplementary Fig. B. Immunofluorescence showed the TFAP2B/COX-2 expression in BCPAP and Nthy-ori-3-1 cell line (Fig. 2b). After TFAP2B antibody incubation, both nucleic and cytoplasmic expression of TFAP2B can be detected in Nthy-ori-3-1 and BCPAP cell line. While BCPAP cell line was more abundant in expression (Fig. 2c). Among the cell lines examined, the expression of TFAP2B/COX-2 was obviously higher in thyroid cancer cell lines than in normal human thyroid cell lines.

As the differential expression of TFAP2B/COX-2 in Nthy-ori-3-1 cell line and thyroid cancer cell lines was shown above, we also wanted to know whether the expression of TFAP2B/COX-2 would be different between thyroid cancer patient tissue samples and the corresponding normal thyroid tissues. Here, we collected 252 thyroid cancer patient thyroid tumor tissues and the corresponding normal thyroid tissues. As the immunohistochemical results shown in Fig. 2d, the results in Fig. 2d was quantified and statistical analyzed (Supplementary Table), we found that the protein levels of TFAP2B and COX-2 were significantly higher in thyroid cancer tissues than in the adjacent tissues, as well as western blot results (Fig. 2e), and the relative protein level is shown in Supplementary Fig. A. Meanwhile, the expression of TFAP2B was positively correlated with the expression of COX-2 (*P* < 0.05) (Fig. 2f).

### High TFAP2B and COX-1 expression is associated with aggressive clinicopathological features

To further investigate the correlation between TFAP2B and COX-2 expression and clinicopathological features, immunohistochemical (IHC) analyses were performed using 252 PPFE PTC samples. The scoring results of the IHC experiments are shown in Table 2. Tumors with scores of ≥ 2 points were considered to have high expression levels, and those with scores of 1 point were categorized into the group with medium expression levels. Scores of zero were categorized into the group with low expression levels. Our study showed that sex, age, thyroid cancer-assisted Hashimoto’s thyroiditis (HT), and recurrence did not have any statistically significant difference according to TFAP2B/COX-2 expression levels (*P* > 0.05). However, high TFAP2B/COX-2 expression levels were associated with multifocal thyroid cancer and N stage (*P* < 0.05). TFAP2B expression was also significantly associated with T/M staging (*P* < 0.05). In contrast to TFAP2B, COX-2 expression was significantly associated with extrathyroidal extension (*P* < 0.05) (Table 2). As the results are shown above, we found that TFAP2B/COX-2 expression is likely connected with invasion and metastasis.

**Table 2:** TFAP2B/COX-2 correlated with clinicopathological characteristics in thyroid cancer

Variable	TFAP2B			*P*	COX-2			*P*
	Low	Medium	High		Low	Medium	High	
Age								
≤55	83	76	38	0.490	52	84	61	0.213
>55	19	22	14		12	19	24	
Gender								
Male	37	35	17	0.903	23	37	29	0.960
Female	65	63	35		41	66	56	
T classification								
I	60	36	11	**0.000**	33	44	30	0.666
II	31	39	19		19	37	33	
III	9	17	15		9	16	16	
IV	2	6	7		3	6	6	
N classification								
N0	45	29	13	**0.026**	29	25	33	**0.012**
Nx	57	69	39		35	78	52	
M classification								
M0	102	95	49	**0.050**	63	100	83	0.676
M1	0	2	3		1	3	1	
Multifocal thyroid cancer								
Yes	21	18	20	**0.015**	8	24	27	**0.023**
No	81	80	32		56	79	58	
Extrathyroidal extension								
Yes	12	19	13	0.100	7	14	23	**0.015**
No	90	79	39		57	89	62	
Thyroid cancer-assisted HT								
Yes	19	20	12	0.808	11	27	13	0.140
No	83	78	40		53	76	72	
Recurrence								
Yes	54	53	30	0.853	32	60	45	0.552
No	48	45	22		32	43	40	

*HT* Hashimoto’s thyroiditis

High TFAP2B/COX-2 expression levels were associated with multifocal thyroid cancer and N stage (*P* < 0.05). TFAP2B expression was also significantly associated with T/M staging (*P* < 0.05). In contrast to TFAP2B, COX-2 expression was significantly associated with extrathyroidal extension (*P* < 0.05). As the results are shown above, we found that TFAP2B/COX-2 expression is likely connected with invasion and metastasis in thyroid cancer

Bold values indicated statistic significance

### TFAP2B promoted thyroid cancer cell growth and proliferation via the COX-2 signaling pathway in vitro

**Fig. 3:**
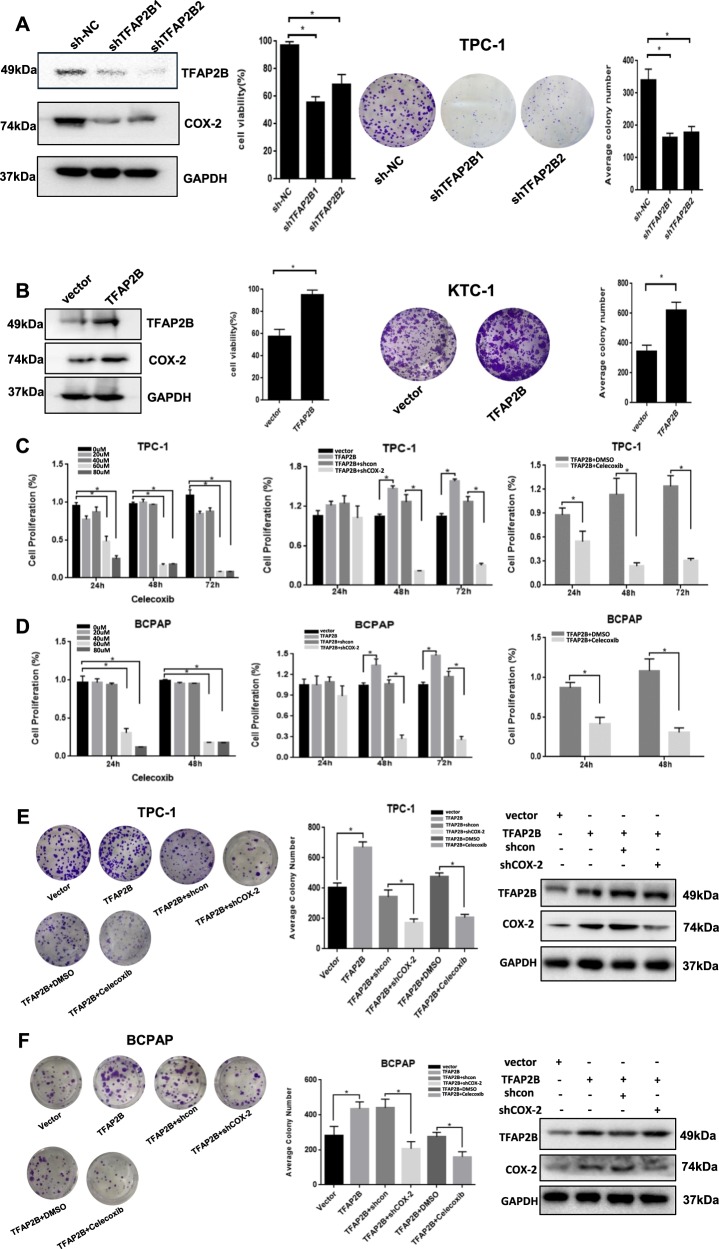
TFAP2B promoted thyroid cancer cell proliferation and clonogenicity via the COX-2 signaling pathway in vitro. **a** Cell proliferation and clonogenicity were suppressed by downregulating TFAP2B in TPC-1 cell line. **b** Cell proliferation and clonogenicity were promoted by upregulating TFAP2B in KTC-1 cell line. **c**, **d** Cell proliferation was suppressed by COX-2 inhibitor celecoxib; overexpression of TFAP2B promoted the expression of COX-2 and increased thyroid cancer cell proliferation, which was reversed by COX-2 knockdown or COX-2 inhibitor celecoxib in TPC-1 and BCPAP cell line. *P < 0.05 (*n* = 3). **e**, **f** Overexpression of TFAP2B promoted the thyroid cancer cell clonogenicity, which was reversed by COX-2 knockdown or COX-2 inhibitor celecoxib in TPC-1 and BCPAP cell line. *P < 0.05 (*n* = 3)

We established TFAP2B knockdown and overexpression stable thyroid cancer cell lines to further investigate the function of TFAP2B in thyroid cancer cell growth and proliferation in vitro and in vivo. We found that thyroid cancer cell viability and colony formation were inhibited in the knockdown TFAP2B cell line (Fig. 3a). Meanwhile, overexpression of TFAP2B significantly promoted thyroid cancer cell viability and colony formation (Fig. 3b). Moreover, this promotion mediated by the overexpression of TFAP2B was reversed by the COX-2 inhibitor Celecoxib, and downregulate COX-2 expression rescued the TFAP2B overexpression experiments in the TPC-1 cell line and the BCPAP cell line (Fig. 3c–f). The above results indicated that TFAP2B could promote thyroid cancer cell growth and proliferation via the COX-2 signaling pathway in vitro.

### TFAP2B promoted thyroid cancer cell growth via the COX-2 signaling pathway in vivo

**Fig. 4:**
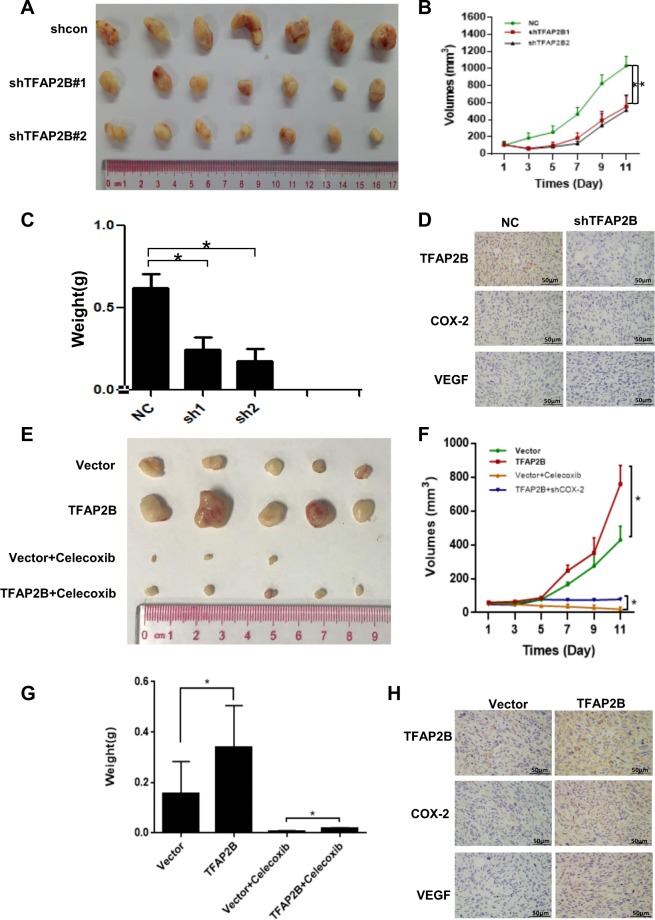
TFAP2B promoted thyroid cancer cell growth via the COX-2 signaling pathway in vivo. **a** TFAP2B knockdown suppressed thyroid cancer cell growth in a mouse xenograft. Images of the thyroid cancer cell tumor xenograft from each mouse (*n* = 7 mice/group). **e** TFAP2B overexpression promoted thyroid cancer cell growth in a mouse xenograft, but suppressed by COX-2 inhibitor celecoxib when feeded to mice. Images of the thyroid cancer cell tumor xenograft from each mouse (*n* = 5 mice/group). **b**, **f** Tumor volumes were analyzed. *P < 0.05. **c**, **g** Tumor weights were recorded and analyzed. *P < 0.05. **d**, **h** The expression of TFAP2B and COX-2 in tumor tissues was analyzed by immunohistochemistry (IHC) staining

Furthermore, we established a thyroid cancer xenograft model in nude mice to demonstrate that TFAP2B can regulate thyroid cancer cell growth in vivo, we injected nude mice with thyroid cancer cells stably expressing TFAP2B-shRNAs or the control shRNAs and stably expressing TFAP2B or the control vector. We also fed celecoxib to TFAP2B-overexpression nude mice to establish a rescue model at the same time. Tumor growth was monitored over a period of nearly 3 weeks. Tumors in the TFAP2B knockdown mice grew slower than those in the mice with the control shRNA (Fig. 4a–c). In contrast, TFAP2B-overexpressing xenografts grew more rapidly in size and weight than the controls, but xenografts with TFAP2B overexpression and COX-2 inhibitor had slower growth in size and weight than those with TFAP2B overexpression without COX-2 inhibitor (Fig. 4e–g). The expression of TFAP2B and COX-2 in tumor tissues was analyzed by IHC staining (Fig. 4d–h). The body weight and health conditions of these nude mice were not affected after all any of these treatments (data not shown). In conclusion, these results indicated that TFAPB promoted thyroid cancer cell growth via COX-2 in vitro and in vivo.

### TFAP2B regulates thyroid cancer cell migration, invasion, and apoptosis via the COX-2 signaling pathway in vitro

**Fig. 5:**
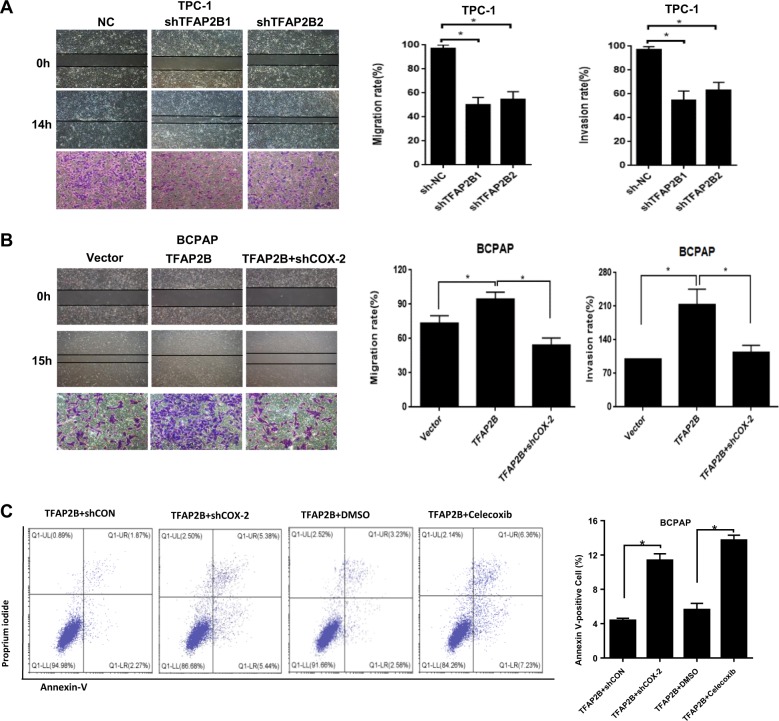
TFAP2B regulates thyroid cancer cell migration, invasion and cell apoptosis via the COX-2 signaling pathway in vitro. **a** Knockdown of TFAP2B inhibited the cell migration and invasion in TPC-1 cells. **b** Knockdown of COX-2 reversed the promotion of cell migration and invasion by TFAP2B overexpression in BCPAP cells. **c** Cell apoptosis was measured by Annexin-V/PI assays in TFAP2B overexpression BCPAP cells (with or without COX-2 knockdown, 60 µM celecoxib, *n* = 3), histograms indicated subpopulation of cells positive for Annexin V/PI

In this study, we detected the function of TFAP2B in the processes of thyroid cancer cell migration and invasion using wound-healing and transwell-invasion assays. After we knockdown TFAP2B in TPC-1 cell lines, the migration and invasion rates obviously decreased (Fig. 5a). Overexpression of TFAP2B in BCPAP cell line increased the migration and invasion rates (Fig. 5b).

Moreover, knockdown of COX-2 in TFAP2B-overexpressing cells decreased the migration and invasion rates when compared with the TFAP2B-overexpressing cells, with the control vector (Fig. 5b). In the cell-apoptosis analysis, we observed that TFAP2B-overexpressing stable cell lines with COX-2-knockdown obviously increased cell death rate compared with the negative control cells. Celecoxib had the same effect (Fig. 5c).

### TFAP2B regulates COX-2 effects via the VEGF/PEDF signaling pathway

**Fig. 6:**
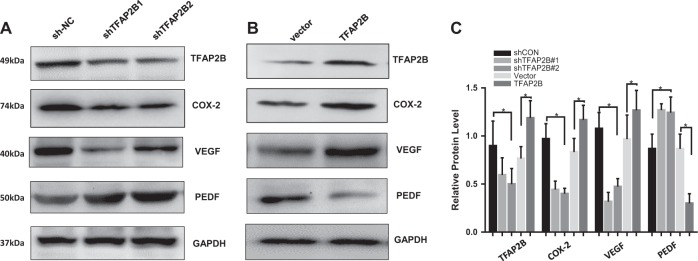
TFAP2B regulates COX-2 effects via the VEGF/PEDF signaling pathway. **a** Knockdown of TFAP2B dramatically inhibited the expression of VEGF proteins and increased the expression of PEDF protein, and **b** the overexpression of TFAP2B resulted in the opposite results. **c** Quantification for the relevant expression level of proteins was also analyzed

The VEGF/PEDF signaling pathway-mediated angiogenesis plays an important role in tumor growth. Previously, researchers have been reported that PGE2 may be an important mediator between COX-2 and VEGF expression in the process of angiogenesis in pancreatic cancer and colorectal cancer^24–26^. We next determined the effect of TFAP2B/COX-2 on the expression of VEGF and PEDF proteins. Knockdown of TFAP2B dramatically inhibited the expression of VEGF proteins and increased the expression of PEDF proteins (Fig. 6a), and the overexpression of TFAP2B resulted in the opposite results (Fig. 6b). The relative protein level is shown in Fig. 6c. These results indicate that TFAP2B regulates COX-2 effects the VEGF/PEDF signaling pathway.

## Discussion

Papillary thyroid cancer (PTC) accounts for ~80% of all thyroid cancers^27,28^. Although thyroid cancer generally has a favorable prognosis, some patients still develop local recurrence with or without distant metastases or cervical lymph node metastasis and die from this disease. Therefore, it is necessary for us to find a biomarker that is an effective diagnostic and therapeutic target for thyroid cancer, especially for refractory thyroid cancer.

Resent studies showed that TFAP2B as one of the corneal endothelial-specific transcription factors^29^, and identified ZP4 as a target gene of TFAP2B, TFAP2B could bind to the ZP4 promoter region and directly induce the expression of the ZP4 protein^30^. In our study, TFAP2B expression was high in papillary thyroid cancer cell lines compared with normal cells and in tumor tissues compared with tissues adjacent to tumors. To confirm our conjecture that TFAP2B acts in thyroid cancer tumorigenesis and development, we knocked down TFAP2B in thyroid cancer cell lines and found that cell viability, clonogenicity, migration, and invasion were reduced in vitro, and the opposite effect occurred after overexpression of TFAP2B in these thyroid cancer cells. In this study, we verified that TFAP2B regulates tumor growth in a cancer xenograft mouse model in vivo.

TFAP2B can specifically bind to the COX-2 promoter in thyroid cancer cells. Our study also showed that COX-2 expression was regulated at the RNA and protein level by TFAP2B. We also found that the acceleration of cell proliferation, apoptosis, migration, and invasion mediated by TFAP2B overexpression could be rescued by COX-2 knockdown and celecoxib. We observed that overexpression or knockdown of TFAP2B can regulate COX-2 expression, thereby impacting VEGF/PEDF signaling. In another study, it was found that quercetin nanoparticles could reduce the expression of TFAP2B and decrease its binding to hTERT promoter to inhibit telomerase reverse transcriptase (hTERT), thus inhibiting NF-κB/COX-2 and impeding Akt/ERK1/2 signaling pathways^31^. The modulation of TFAP2B was also reported in retinogenesis, which suggested that Ptf1a might act upstream of TFAP2A and TFAP2B to activate their expression^32^. Moreover, an epigenetic relationship of TFAP2B was found with metabolic fitness^33^.

In summary, these results suggest that TFAP2B regulates thyroid cancer development and progression via COX-2-mediated downstream signaling pathways. Interestingly, the COX-2 inhibitor celecoxib inhibited thyroid tumor growth in animal experiments. COX-2 plays a role in tumor development and progression. Previous studies have shown that its inhibitors can inhibit breast tumor growth in mice models^34^, but cannot prolong the survival of breast cancer patients^35^. Although thyroid cancer has a good prognosis, recurrence, and metastasis frequently occur, therefore, if COX-2 inhibitors can be applied in clinical practice and inhibit tumor growth in thyroid cancer patients, the medical burden can be significantly relieved.

The clinicopathologic data from our tissue array showed that high expression of TFAP2B/COX-2 is relevant to more advanced T classification and N classification, and more likely to be associated with multifocal carcinoma in thyroid cancer.

COX-2 is associated with the inflammatory response. Thyroid cancer is often accompanied by Hashimoto’s thyroiditis, but there was no statistically significant correlation between COX-2 expression and Hashimoto’s thyroiditis. Whether inflammatory cytokines such as TNF, IL-6, IL-1, et al. are associated with COX-2 expression and affect the recurrence, metastasis, and prognosis still needs further study. The regulatory mechanism of TFAP2B/COX-2 also needs in-depth study.

In conclusion, in the thyroid cancer cell line, COX-2 signaling can be modulated by TFAP2B, and TFAP2B drives tumor development in thyroid cancer as an oncogene. TFAP2B binds to the promoter of COX-2 to activate its expression, revealing that TFAP2B is a ponderable regulatory molecule in the COX-2 signaling pathway, affecting tumor progression in thyroid cancer and is a potentially effective target for thyroid cancer diagnosis and therapy.

## Materials and methods

### Cell lines and cell culture

The normal thyroid epithelial Nthy-ori-3-1 cell line was purchased from ShunRan Biological Technology (Shanghai), and Ktc-1, Bcpap, and Tpc-1 cells were obtained from the Shanghai Institute of Biochemistry and Cell Biology, CAS (Cell Bank/Stem Cell Technology platform). Nthy-ori-3-1, Ktc-1, Bcpap, and TPC-1 cells were cultured in RPMI-1640 (Gibco BRL,Grand Island, NY) supplemented with 10% fetal bovine serum, and 100 units.mL^−1^ penicillin/100 lg.mL^−1^ streptomycin. All cells were cultured in an incubator with a humidified atmosphere with 5% CO_2_ at 37 °C.

### shRNA and stable cell lines

To overexpress TFAP2B in thyroid cancer cells, pcDNA3.1–TFAP2B or control vector plasmids were transfected with EndoFectinTM MAX (GeneCopoeia, Inc., Rockville, MD, USA). To knockdown gene expression, thyroid cancer cells were transfected with specific short hairpin TFAP2B RNA (shRNA, 5′-GGAAGCUUGUGGAGAAUGUTT-3′ and CCCGAAAGAAUAUGCUGUUTT) and COX-2 RNA (shRNA, 5′-AAC UGC UCA ACA CCG GAA Udtdt-3′). The shRNAs were purchased from GenePharma Co., Ltd. (Suzhou, China), and to inhibit COX-2 by the COX-2 inhibitor Celecoxib (purchased from Selleck).

We use KTC-1, Bcpap and TPC-1 cells lines to establish stable cell lines by selection with 1 lg.mL^−1^ puromycin for 4 weeks, and adenoviruses were purchased from GenePharma Co., Ltd.

### Antibodies and western blot analysis

We use 10% SDS–PAGE gels to separated equal amounts of protein and transferred onto PVDF membranes for detection. The membranes were then sequentially incubated with specific antibodies, and the protein bands were detected using enhanced chemiluminescence. Anti-TFAP2B was purchased from Santa Cruz Biotechnology (sc-166441), anti-COX-2 was purchased from Cell Signaling Technology (#12282), and anti-GAPDH (10494-1-AP), anti-VEGF (19003-1-AP), anti-PEDF (26045-1-AP) were purchased from Proteintech (Wuhan, China).

### RNA extraction and quantitative RT-PCR (qRT-PCR)

We extracted the total RNA from cells using a RaPure Total RNA Micro Kit (Magen, Guangzhou, China). A ReverTraAce qPCR RT Master Mix kit (ToYoBo, Shanghai, China) was used to generate endogenous cDNA. The qRT-PCR primers TFAP2B (5′-GCGGCATGAATCTATTGGAC-3′) was purchased from Invitrogen (Shanghai), the following qRT-PCR primers were purchased from GeneCopoeia, Inc. (Rockville, MD, USA): COX-2 (5′-TCACAGGCTTCCATTGACCAG-3′), and GAPDH (HQP006940). qRT-PCR was performed with the SYBR Green Real-time PCR Master Mix (ToYoBo, Shanghai, China).

### Cell proliferation

An MTS assay (Promega Biotech Co., Ltd., Madison, WI, USA) was used to determine the cell viability. Cells were seeded in 96-well plates (6000 cells/well), cultured overnight, and then transfected with TFAP2B shRNA or a negative control. Cell viability was detected 24 h, 48 h, and 72 h after transfection. Cell viability of stable cell lines with TFAP2B overexpression with/without the COX-2 inhibitor celecoxib was detected 48 h after plating in 96-well plates (4000 cells/well). As well as cell viability of stable cell lines with TFAP2B overexpression transfected with a negative control and shCOX-2.

### Cell colony formation

Cells with TFAP2B knockdown or overexpression with/without the COX-2 inhibitor celecoxib were seeded in six-well plates (500 cells/well) and incubated for 14 days. Cells with TFAP2B overexpression were transfected with a negative control and shCOX-2, as well. We used formalin to fix the cells for 10 min and then stained the cells with crystal violet for 15 min. Next, we captured the images of the clones and counted the numbers of the clones using Image-Pro Plus 6.0 software.

### Scratch, transwell invasion, and cell-apoptosis assay

Cells were cultured overnight and then transfected with TFAP2B shRNA and the TFAP2B overexpression plasmid in six-well plates. After 48 h, cells reached a density of 90%, then we used a 100-µL pipette tip to scratch the cell monolayers, and washed with PBS three times to remove the detached cells. We imaged the scratch assay and measured the widths of the gap at 0 h (w1) and 16 h (w2), and we calculated the results ((w1-w2)/w1 × 100%). Cell-invasion assay was performed with BD BioCoat Matrigel Invasion Chambers (Becton Dickinson, Franklin Lakes, NJ) per the manufacturer’s instruction. We counted five random fields under the light microscope for statistics. Annexin V-FITC and PI (4 A Biotech Co. cat. no. FXP018) was used for cell-apoptosis analysis with flow cytometer.

### COX-2 promoter luciferase plasmids and luciferase reporter assay

In order to study the transcriptional regulation of COX-2, we constructed dual fluorescent reporter gene plasmids with COX-2 promoter (Primers used to amplify the COX-2 promoter region: 5′-ACGTGACTTCCTCGACCCTC-3′, 5′-CAGGCGCACAGGTTTCCGCC-3′). We used pGL4.10–Luc2 (Promega, AY738222) plasmid as vector, inserted the the COX-2 promoter fragment to pGL4.10–Luc2 between HindIII and XhoI sites. Bcpap cells and K2 cells were seeded in 24-well plates and cultured overnight to a density of 70–80%, transfected with TFAP2B shRNA and the negative control, as well as transfected with TFAP2B overexpression plasmid and control vector, mediated by EndoFectinTM MAX (GeneCopoeia, Inc., Rockville, MD, USA). After 72 h of cultivation, they were transfected with the COX-2 promoter luciferase plasmids. Thirty-six hours after treatment, the luciferase activity was measured using a luciferase reporter assay kit (Promega Biotech Co., Ltd., Madison, WI, USA).

### Animal experiments

We bought 4-week-old female BALB/c nude mice from Vital River Laboratory Animal Technology Co., Ltd. (Beijing, China) and quarantined them for 1 week before the experiments. We suspended the cells (3 × 10^6^) by 100 µl of PBS, then subcutaneously injected into the BALB/c mice. We measured the weight of the mice and the volume of the tumors every 2 days for nearly 3 weeks. The mice were killed at the end of the experiments, and then we excised the tumors, photographed them, and processed them for immunohistochemical analyses. All animal experiment procedures were approved by the Animal Care and Use Committee of Sun Yat-sen University, and every effort was made to reduce the suffering of animals. The animal experiments strictly followed the ethical guidelines.

### Immunohistochemistry

Tissue microarrays with 252 samples of formalin-fixed paraffin-embedded (FFPE) thyroid cancer and matched adjacent normal tissues were provided by Sun Yat-sen University Cancer Center. The microarrays were incubated with anti-TFAP2B and anti-COX-2 primary antibodies and secondary antibodies, and after color development, scoring was performed.

### Statistical analysis

Statistical analyses were performed using the SPSS statistical software package (version 24.0). The Chi-square test and *t* tests were applied for variance analysis, Spearman’s rank correlation method was used for correlation analysis, and *P* < 0.05 was considered statistically significant.

### Ethics approval

Thyroid cancer tissues were collected from patients who underwent surgical resection at the Sun Yat-sen University Cancer Center Head and Neck Surgery Department (SYSUCC, Guangzhou, China). All patients signed consent letters and all manipulation of the tissues were approved by the Ethics Committee of Sun Yat-sen University. All animal procedures were in accordance with the guidelines of the Institutional Animal Care and Use Committee and the guidelines of the Guangzhou medical University and Sun Yat-sen University.

## Supplementary information

supplementary table

supplementary figure

Supplementary figure legends

## References

[1] Siegel RL, Miller KD, Jemal A (2018). Cancer statistics, 2018. CA Cancer J. Clin..

[2] Davies L, Welch HG (2014). Current thyroid cancer trends in the United States. JAMA Otolaryngol. Head Neck Surg..

[3] Kim YA, Chang M, Park YJ, Kim JE (2012). Detection of survivin and COX-2 in thyroid carcinoma: anaplastic carcinoma shows overexpression of nuclear survivin and low COX-2 expression. Korean J. Pathol..

[4] Wang YX (2017). HPV16 E6 promotes breast cancer proliferation via upregulation of COX-2 expression. Biomed. Res. Int..

[5] Zhang Z (2017). XRCC5 cooperates with p300 to promote cyclooxygenase-2 expression and tumor growth in colon cancers. PLoS One.

[6] Howe LR (2007). Inflammation and breast cancer cyclooxygenase/prostaglandin signaling and breast cancer. Breast Cancer Res..

[7] Fujita H (2002). Cyclooxygenase-2 promotes prostate cancer progression. Prostate.

[8] Leung WK (2003). Cyclooxygenase-2 upregulates vascular endothelial growth factor expression and angiogenesis in human gastric carcinoma. Int. J. Oncol..

[9] Molina MA, Sitja-Arnau M, Lemoine MG, Frazier ML, Sinicrope FA (1999). Increased cyclooxygenase-2 expression in human pancreatic carcinomas and cell lines: growth inhibition by nonsteroidal anti-inflammatory drugs. Cancer Res..

[10] Talar-Wojnarowska R (2011). Role of cyclooxygenase-2 gene polymorphisms in pancreatic carcinogenesis. World J. Gastroenterol..

[11] Yi C (2016). Cleavage and polyadenylation specific factor 4 targets NF-kappaB/cyclooxygenase-2 signaling to promote lung cancer growth and progression. Cancer Lett.

[12] Ji B, Liu Y, Zhang P, Wang Y, Wang G (2012). COX-2 expression and tumor angiogenesis in thyroid carcinoma patients among northeast Chinese population-result of a single-center study. Int. J. Med. Sci..

[13] Siironen P (2006). VEGF-C and COX-2 expression in papillary thyroid cancer. Endocr. Relat. Cancer.

[14] Eckert D, Buhl S, Weber S, Jager R, Schorle H (2005). The AP-2 family of transcription factors. Genome Biol.

[15] Xu X (2017). AP-2alpha and AP-2beta regulate dorsal interneuron specification in the spinal cord. Neuroscience.

[16] Moser M (1997). Enhanced apoptotic cell death of renal epithelial cells in mice lacking transcription factor AP-2beta. Genes Dev..

[17] Nyboe D (2018). A study of familial Char syndrome involving the TFAP2B gene with a focus on facial shape characteristics. Clin. Dysmorphol..

[18] Satoda M (2000). Mutations in TFAP2B cause Char syndrome, a familial form of patent ductus arteriosus. Nat Genet.

[19] Pellikainen JM, Kosma VM (2007). Activator protein-2 in carcinogenesis with a special reference to breast cancer–a mini review. Int. J. Cancer.

[20] Fu L (2014). TFAP2B overexpression contributes to tumor growth and a poor prognosis of human lung adenocarcinoma through modulation of ERK and VEGF/PEDF signaling. Mol. Cancer.

[21] Wu H, Zhang J (2018). Decreased expression of TFAP2B in endometrial cancer predicts poor prognosis: a study based on TCGA data. Gynecol Oncol.

[22] Raap M (2017). Lobular carcinoma in situ and invasive lobular breast cancer are characterized by enhanced expression of transcription factor AP-2β. Lab. Invest..

[23] Xiao Y (2015). Ku80 cooperates with CBP to promote COX-2 expression and tumor growth. Oncotarget.

[24] Xie C (2018). Cyclooxygenase-2 induces angiogenesis in pancreatic cancer mediated by prostaglandin E2. Oncol Lett.

[25] Zhang Y (2018). Cyclooxygenase-2 inhibition potentiates the efficacy of vascular endothelial growth factor blockade and promotes an immune stimulatory microenvironment in preclinical models of pancreatic cancer. Mol. Cancer Res.

[26] Gungor H, Ilhan N, Eroksuz H (2018). The effectiveness of cyclooxygenase-2 inhibitors and evaluation of angiogenesis in the model of experimental colorectal cancer. Biomed. Pharmacother.

[27] Jiang C (2018). Clinical behaviors of rare variants of papillary thyroid carcinoma are associated with survival: a population-level analysis. Cancer Manag. Res..

[28] Selemetjev S, Savin S, Paunovic I, Tatic S, Cvejic D (2018). Concomitant high expression of survivin and vascular endothelial growth factor-C is strongly associated with metastatic status of lymph nodes in papillary thyroid carcinoma. J. Cancer Res. Ther..

[29] Yoshihara M (2017). Restricted presence of POU6F2 in human corneal endothelial cells uncovered by extension of the promoter-level expression atlas. EBioMedicine.

[30] Hara S (2019). Transcription factor TFAP2B up-regulates human corneal endothelial cell-specific genes during corneal development and maintenance. J. Biol. Chem..

[31] Ren KW (2017). Quercetin nanoparticles display antitumor activity via proliferation inhibition and apoptosis induction in liver cancer cells. Int. J. Oncol..

[32] Jin K (2015). Tfap2a and 2b act downstream of Ptf1a to promote amacrine cell differentiation during retinogenesis. Mol. Brain.

[33] Caspers M (2019). Metabolic fitness in relation to genetic variation and leukocyte DNA methylation. Physiol Genomics.

[34] Krall Jordan A., Reinhardt Ferenc, Mercury Oblaise A., Pattabiraman Diwakar R., Brooks Mary W., Dougan Michael, Lambert Arthur W., Bierie Brian, Ploegh Hidde L., Dougan Stephanie K., Weinberg Robert A. (2018). The systemic response to surgery triggers the outgrowth of distant immune-controlled tumors in mouse models of dormancy. Science Translational Medicine.

[35] Strasser-Weippl K (2018). Effects of celecoxib and low-dose aspirin on outcomes in adjuvant aromatase inhibitor-treated patients: CCTG MA.27. J. Natl. Cancer Inst..

[36] Wasserman WW, Sandelin A (2004). Applied bioinformatics for the identification of regulatory elements. Nat. Rev. Genet..

