# Supplementary material for: TFAP2B overexpression contributes to tumor growth and progression of thyroid cancer through the COX-2 signaling pathway
Source: Cell Death Dis. 2019 May 21;10(6):397. doi: 10.1038/s41419-019-1600-7 (PMC6529436; doi:10.1038/s41419-019-1600-7)
Supplement: Supplementary file 1 — supplementary table [file 41419_2019_1600_MOESM1_ESM.docx]

| **Table 2：Statistic analysis of TFAP2B/COX-2 expression in TCTs and ANTs.** | | | | | | | | | |
| --- | --- | --- | --- | --- | --- | --- | --- | --- | --- |
| **Variable** | **TFAP2B** | | | ***P*** | **COX-2** | | | ***P*** |  |
|  | **Low** | **Medium** | **High** |  | **Low** | **Medium** | **High** |  |  |
| **TCT** | **102** | **98** | **52** |  | **64** | **103** | **85** |  |  |
| **ANT** | **143** | **78** | **31** | **＜0.05** | **133** | **63** | **56** | **＜0.05** |  |

**(TCT: thyroid cancer tissue, ANT:** **adjacent non-carcinoma tissues)**
