# Supplementary figures and images for: TFAP2B overexpression contributes to tumor growth and progression of thyroid cancer through the COX-2 signaling pathway
Source: Cell Death Dis. 2019 May 21;10(6):397. doi: 10.1038/s41419-019-1600-7 (PMC6529436; doi:10.1038/s41419-019-1600-7)

A

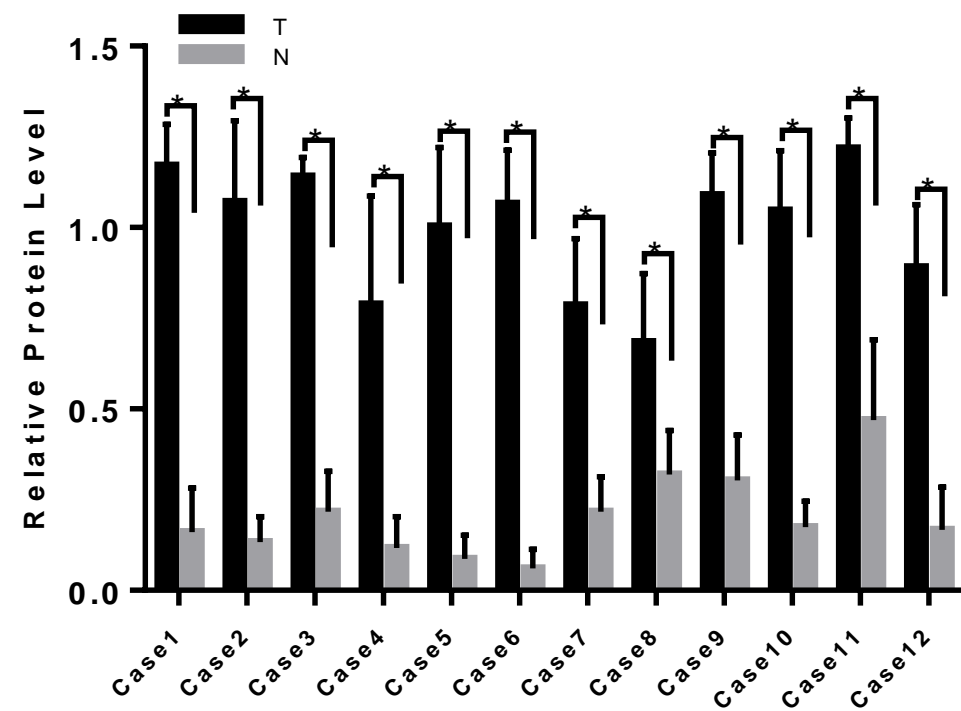

B

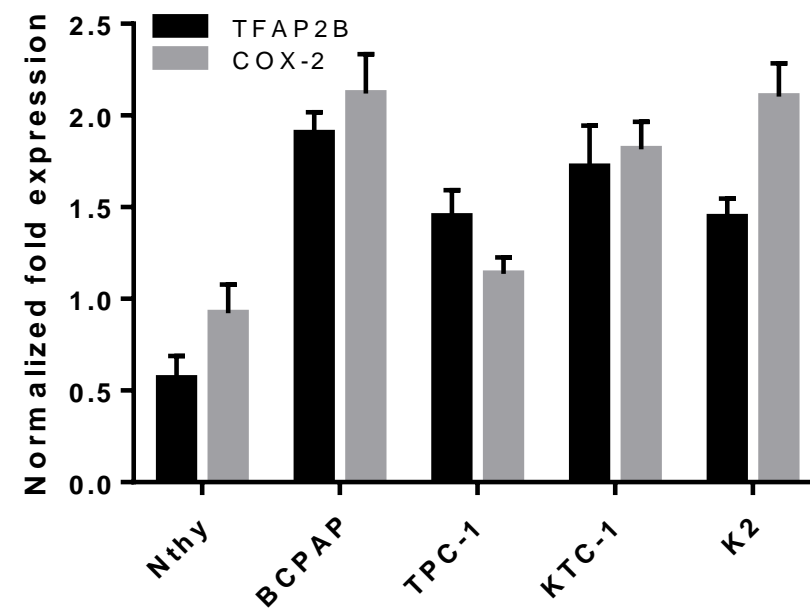

Supplement: Supplementary file 2 — supplementary figure [file 41419_2019_1600_MOESM2_ESM.pdf]
