# Supplementary material for: TFAP2B overexpression contributes to tumor growth and progression of thyroid cancer through the COX-2 signaling pathway
Source: Cell Death Dis. 2019 May 21;10(6):397. doi: 10.1038/s41419-019-1600-7 (PMC6529436; doi:10.1038/s41419-019-1600-7)
Supplement: Supplementary file 3 — Supplementary figure legends [file 41419_2019_1600_MOESM3_ESM.docx]

**Supplymentary Figure:** The relative protein levels of TFAP2B/COX-2 in Thyroid Cancer tissues is higher than ANTs.

**Supplymentary Table：**The expression of TFAP2B/COX-2 in Thyroid Cancer tissues is higher than ANTs.
